# Controlled Growth of Large-Area Aligned Single-Crystalline Organic Nanoribbon Arrays for Transistors and Light-Emitting Diodes Driving

**DOI:** 10.1007/s40820-017-0153-5

**Published:** 2017-08-16

**Authors:** Wei Wang, Liang Wang, Gaole Dai, Wei Deng, Xiujuan Zhang, Jiansheng Jie, Xiaohong Zhang

**Affiliations:** Jiangsu Key Laboratory for Carbon-Based Functional Materials and Devices, Institute of Functional Nano and Soft Materials (FUNSOM), Soochow University Suzhou, Suzhou, 215123 Jiangsu People’s Republic of China

**Keywords:** Large-area growth, Organic single-crystalline nanoribbon arrays, Organic field-effect transistors, Light-emitting diodes driving

## Abstract

**Abstract:**

Organic field-effect transistors (OFETs) based on organic micro-/nanocrystals have been widely reported with charge carrier mobility exceeding 1.0 cm^2^ V^−1^ s^−1^, demonstrating great potential for high-performance, low-cost organic electronic applications. However, fabrication of large-area organic micro-/nanocrystal arrays with consistent crystal growth direction has posed a significant technical challenge. Here, we describe a solution-processed dip-coating technique to grow large-area, aligned 9,10-bis(phenylethynyl) anthracene (BPEA) and 6,13-bis(triisopropylsilylethynyl) pentacene (TIPS-PEN) single-crystalline nanoribbon arrays. The method is scalable to a 5 × 10 cm^2^ wafer substrate, with around 60% of the wafer surface covered by aligned crystals. The quality of crystals can be easily controlled by tuning the dip-coating speed. Furthermore, OFETs based on well-aligned BPEA and TIPS-PEN single-crystalline nanoribbons were constructed. By optimizing channel lengths and using appropriate metallic electrodes, the BPEA and TIPS-PEN-based OFETs showed hole mobility exceeding 2.0 cm^2^ V^−1^ s^−1^ (average mobility 1.2 cm^2^ V^−1^ s^−1^) and 3.0 cm^2^ V^−1^ s^−1^ (average mobility 2.0 cm^2^ V^−1^ s^−1^), respectively. They both have a high on/off ratio (*I*_on_/*I*_off_) > 10^9^. The performance can well satisfy the requirements for light-emitting diodes driving.

**Graphical Abstract:**

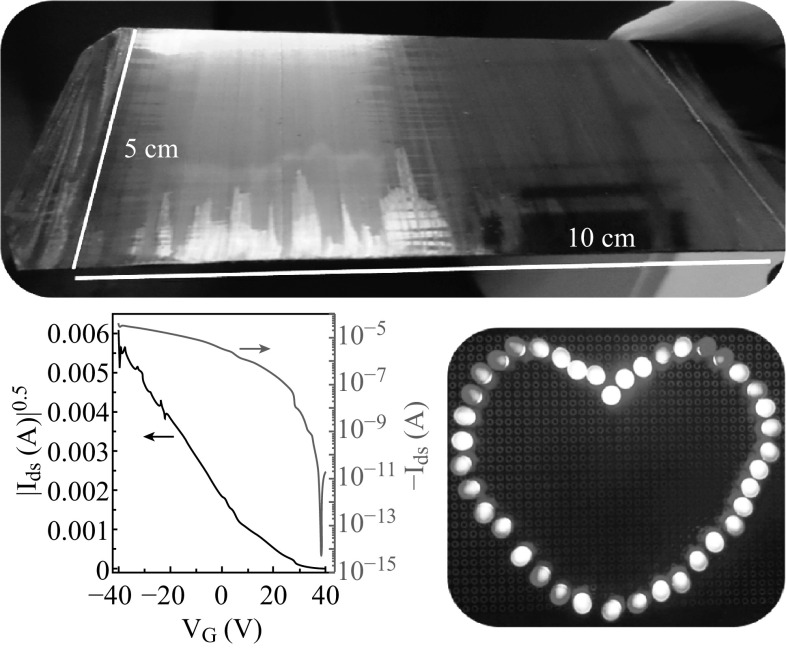

**Electronic supplementary material:**

The online version of this article (doi:10.1007/s40820-017-0153-5) contains supplementary material, which is available to authorized users.

## Highlights

- A simple solution-processed dip-coating method achieves large-area single-crystalline nanoribbon arrays (5 × 10 cm^2^). High-performance organic field-effect transistors (OFETs) can be obtained through this method.
- Organic nanoribbon array-based OFETs exhibit long-time cycle stability, enabling the control of light emission of different pixel patterns of LEDs.

## Introduction

Single-crystalline organic micro-/nanocrystals have attracted considerable attention in the past decade due to their superior optical and electronic properties, such as high mobilities, long-range diffusion of triplet excitons, and high luminous efficiencies [1–3]. They have found important applications in diverse fields, including organic field-effect transistors (OFETs), photodetectors (PDs), organic light-emitting diodes (OLEDs), and sensors [4–12]. For instance, Hu et al. reported the fabrication of high-performance p-channel OFETs based on the small-molecule organic crystal; the p-OFETs based on dibenzo[d, d′]thieno[3,2-b;4,5-b′]dithiophenes (C6-DBTDT) nanometer-sized ribbons showed mobility higher than 18.9 cm^2^ V^−1^ s^−1^ [13]. Zhang et al. [14] introduced a Schottky-type junction structure in 2,4-bis[4-(N,N-dimethylamino)phenyl]squaraine (SQ) nanowire-based devices to achieve high-sensitivity photodetection. High-brightness OLEDs were achieved using high-mobility and high-photoluminescence quantum yield organic single crystals [15]. These devices exhibit superior performance compared to their counterparts fabricated from conventional organic films, owing to perfect order of molecules and absence of grain boundaries in organic micro-/nanocrystals.

Up to now, most of OFETs based on single-crystalline organic micro-/nanocrystals demonstrate mobility over 1.0 cm^2^ V^−1^ s^−1^ [6, 13, 16, 17]. This value has been surpassing that of amorphous silicon (*α*-Si)-based field-effect transistors (FETs) (mobility of 0.1–1.0 cm^2^ V^−1^ s^−1^ and on/off ratios of 10^6^–10^8^) and approaching that of polycrystalline silicon (*c*-Si)-based FETs (mobility larger than 10 cm^2^ V^−1^ s^−1^) [16–21], revealing the great potential of the organic micro-/nanocrystals for high-performance, low-cost organic electronics. Despite this progress, rational control of crystal orientations and growth directions of the organic micro-/nanocrystals in large area has posed significant technical challenges [22, 23]. Alignment and patterning of the organic micro-/nanocrystals can reduce or eliminate parasitic leakage paths, improve device uniformity and reproducibility, and thus facilitate the device fabrication and integration. Also, as a result of the anisotropic nature of charge transport in organic micro-/nanocrystals, control on the azimuthal orientation of the crystals in a desirable direction (*π*–*π* stacking direction in general) is critical to the optimal charge transport of the devices [24]. To date, a variety of deposition techniques have been developed for the aligned growth of single-crystalline organic micro-/nanocrystals [22–29], such as droplet-pinned crystallization (DPC) [22], geometry-restricted evaporation [25], and direct printing [28, 29]. However, these fabrication methods usually require growth templates, complex instruments, or multi-step processes to obtain aligned organic micro-/nanocrystals [25, 29] and are not quite suitable for convenient, large-area commercial productions. So, a new deposition technique is surely desired to satisfy the practical applications.

9,10-Bis(phenylethynyl) anthracene (BPEA) and 6,13-bis(triisopropylsilylethynyl) pentacene (TIPS-PEN) are well known for excellent electrical characteristics due to their strong intermolecular π-π interaction and have been broadly used in OFETs [30, 31]. However, the spun BPEA and TIPS-PEN films generally display a low crystallinity and poor OFET performances (with mobility below 0.1 cm^2^ V^−1^ s^−1^). Recent studies demonstrated that the single-crystalline BPEA and TIPS-PEN without any defects and grain boundary can improve OFET performances [30]. However, the typical device area was very small, which is not capable of producing a high density of device with reasonable throughput.

Herein, we report a facile dip-coating method for large-area deposition of well-aligned single-crystalline BPEA and TIPS-PEN nanoribbon arrays. The quality of organic nanocrystals was controlled by tuning the dip-coating speed. Moreover, OFETs based on the organic crystal arrays were systematically investigated. Our work is expected to have a great potential of the aligned single-crystalline organic nanoribbons for high-performance, low-cost organic devices.

## Experimental Details

### Substrate Treatment

The silicon wafers were initially cleaned by the chemical cleaning process in a piranha solution (4:1 mixture of H_2_SO_4_:H_2_O_2_) for 10–15 min. The substrates were rinsed several times in deionized water (resistivity = 18 MΩ cm), then dried with a stream of nitrogen, and cleaned with an oxygen plasma (PVA TePla Ion 40) cleaner (200 mm HgO_2_, 300 W) for 600 s in the subsequent procedures.

### Materials and Sample Preparation

Highly doped n-type silicon wafers (resistivity <0.01 Ω cm) with a 300-nm thermally grown silicon oxide gate dielectric layer were used as the substrates for OFET fabrication. The BPEA (received from Sigma-Aldrich) and TIPS-PEN (received from Luminescence Technology Corp.) were used without further purification. The BPEA and TIPS-PEN solutions were both prepared at a concentration of 4 mg mL^−1^ in dichloromethane. The substrate was dipped into a BPEA or TIPS-PEN solution and then lifted out at a constant rate of 10, 30, 60, 80, and 120 μm s^−1^, respectively. Dip coating was performed in clean bench for reducing the effect of air current and mechanical vibration. Electrodes were formed by thermal evaporation using a shadow mask on the active layer.

### Characterizations

The samples were characterized with the assistance of fluorescence microscope (Leica, DM4000M), atomic force microscope (AFM, Veeco MultiMode V), and scanning electron microscope (SEM, FEI Quanta 200 FEG) operated at 20 kV. The crystallinity of the nanoribbon arrays was determined by selective-area electron diffraction (SAED) in transmission electron microscope (TEM, FEI, Tecnai G2 F20) operating at 200 kV and confirmed by X-ray diffraction (XRD, PANalytical BV Empyrean), using a Cu source running at 40 kV and 40 mA. Source and drain electrodes were deposited by thermal evaporation onto the single-crystalline organic nanoribbon arrays through shadow masks that consists of tungsten wires with different diameters, creating transistors with different channel lengths (*L*). Electrical characteristics of the OFETs were measured with a semiconductor parameter analyzer (Keithley 4200-SCS) in air ambient (relative humidity ≈ 30%) at room temperature. The field-effect mobility *μ* and threshold voltage *V*_T_ were calculated in the saturation regime (*V*_DS_ = −50 V) by plotting the square root of the drain current versus the gate voltage using *I*_DS_ = (*W*/2*L*) *C*_i_ *μ* (*V*_G_−*V*_T_)^2^, where *C*_i_ is the capacitance/unit area of the gate dielectric layer, and *W* and *L* are the actual crystal width and channel length, respectively, which were measured using an optical microscope (BX51, Olympus).

## Results and Discussion

### Fabrication of Large-Area, Aligned Single-Crystalline Organic Nanoribbon Arrays

**Fig. 1:**
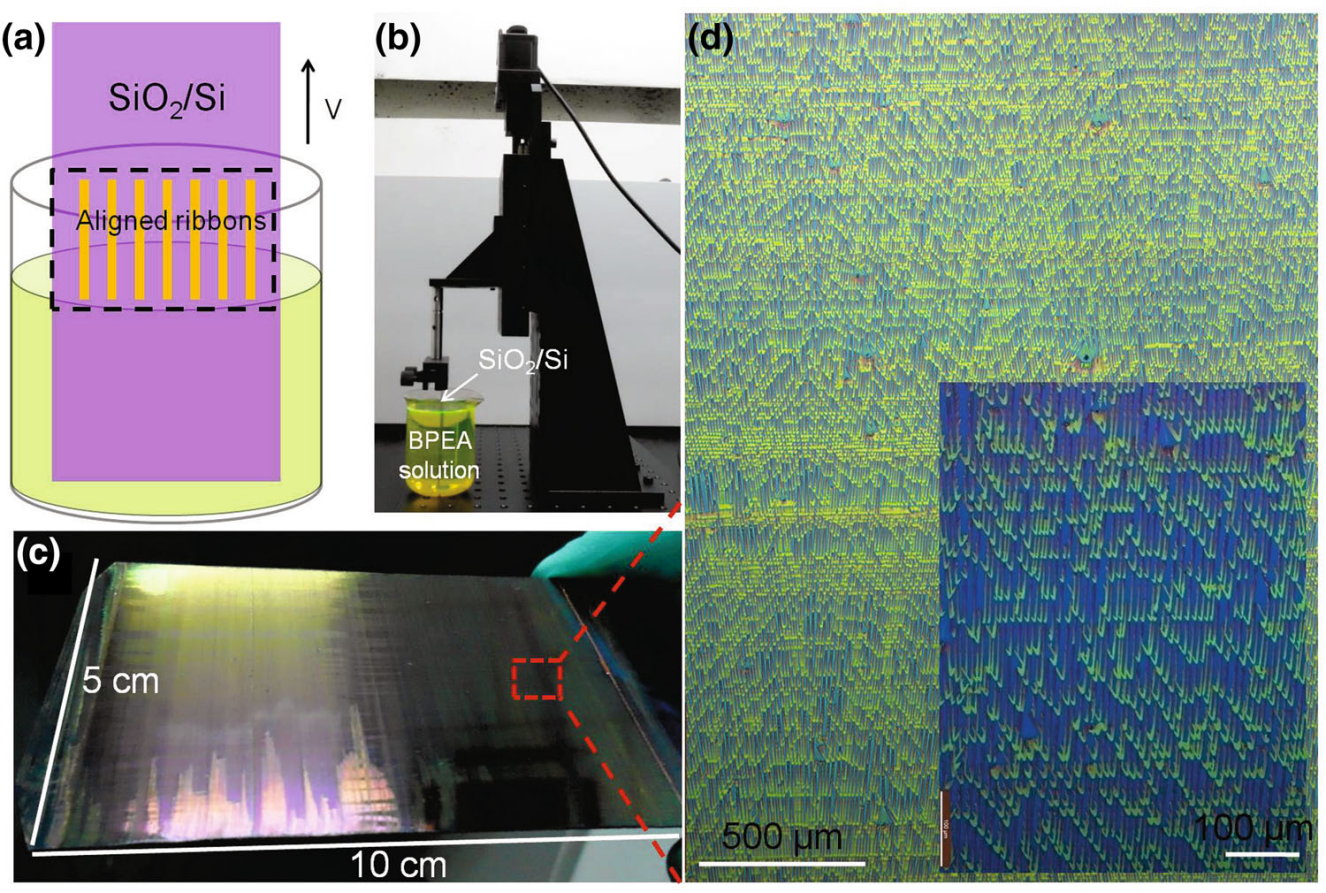
Fabrication of large-area, single-crystalline organic nanoribbon arrays. **a** Schematic illustration of the procedure to fabricate BPEA nanoribbon arrays on the SiO_2_/Si substrate using dip-coating method. **b** Photograph of the dip-coating equipment. **c** 5 × 10 cm^2^ SiO_2_/Si substrate coated with BPEA nanoribbon arrays. **d** Optical microscope image of the large-area BPEA nanoribbon arrays on SiO_2_/Si substrate. *Inset* shows the corresponding enlarged optical microscope image

To demonstrate the efficiency of the dip-coating method for large-area growth of aligned micro-/nanocrystal arrays, BPEA and TIPS-PEN were chosen as model organic semiconductors, because of their high charge carrier mobilities, and have been broadly used in film-based OFETs [32–37]. The dip-coating method used to prepare large-area single-crystalline organic nanoribbon arrays is illustrated in Fig. 1a, b. Firstly, a piece of SiO_2_/Si substrate was immersed vertically into BPEA or TIPS-PEN solution at room temperature; then, the substrate was lifted at a certain coating speed (*V*). With the gradual evaporation of dichloromethane, parallel organic nanoribbons would be deposited along the lifting direction. Figure 1c shows the optical image of the large-sized SiO_2_/Si substrate (5 × 10 cm^2^) coated with BPEA nanoribbon arrays. Notably, the parallel and highly aligned nanoribbon arrays can extend over almost the entire substrate, forming continuous and aligned nanoribbon from top to bottom, as shown in Fig. 1d. The surface coverage of the organic nanoribbon on the substrate is estimated to be around 60% from the optical image.

### Crystal Structures of Aligned Organic Nanoribbons

**Fig. 2:**
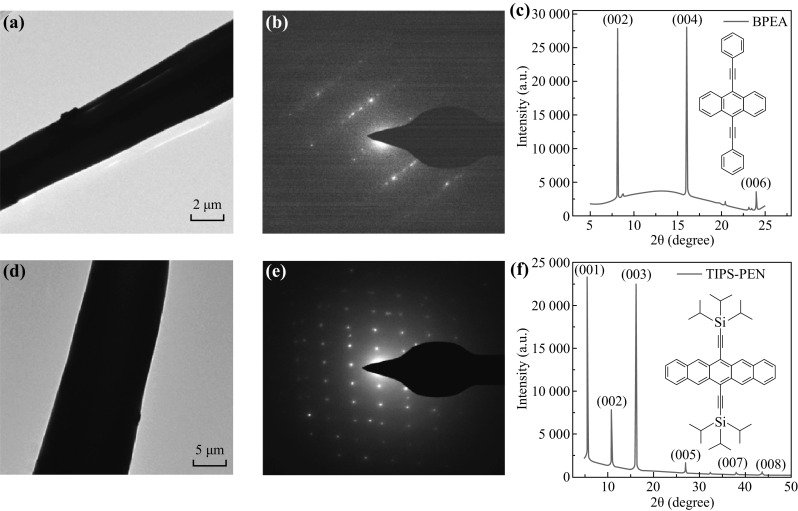
TEM images of the BPEA nanoribbon (**a**) and TIPS-PEN nanoribbon (**d**). **b**, **e** Show the corresponding SAED patterns. **c**, **f** XRD patterns of BPEA and TIPS-PEN nanoribbon arrays fabricated on the SiO_2_/Si substrates, respectively. *Insets* show the molecular structures of BPEA and TIPS-PEN

The structures of the samples were examined by TEM and XRD. TEM images and corresponding SAED patterns of the BPEA and TIPS-PEN nanoribbons are shown in Fig. 2. The presence of discrete diffraction points (Fig. 2b, e) clearly indicates the single-crystalline nature of the BPEA and TIPS-PEN nanoribbon arrays. The nanoribbons have a growth orientation along [010] direction, which coincides with the *π*–*π* stacking directions of BPEA and TIPS-PEN molecules [32, 34]. XRD patterns of the BPEA and TIPS-PEN nanoribbon arrays disclose a well-defined set of (001) reflections (Fig. 2c, f). For the BPEA nanoribbon arrays, the primary peak displays strong diffraction with *d*-spacing of 21.8 Å, which is close to the BPEA *c*-axis length of 22.7 Å calculated by HyperChem 7.0 [34]. As for the TIPS-PEN nanoribbon arrays, the strong and sharp XRD diffraction peak observed at 5.53° suggests a well-organized molecular structure with an interplanar *d*-spacing of 16.1 Å. This value of the *c*-axis length is also approximate to the value derived from single-crystalline data (16.8 Å) [38]. These results collectively demonstrate that large-area growth of single-crystalline organic nanoribbons has been successfully achieved by dip-coating method. Furthermore, 2,7-didecylbenzothienobenzothiophene (C10-BTBT) was also used to fabricate the single-crystalline nanoribbons arrays (Fig. S1), which indicates the good universality of the dip-coating method.

### Morphology Control of Single-Crystalline Organic Nanoribbon Arrays

During the process of dip coating, crystallization of organic molecules is an evaporation-induced procedure, which generates at three-phase contact line. Molecules at the meniscus profile deposit firstly with solvent evaporation. Afterward, the convection flow and capillary force induce molecules from internal solution to flow outward refilling the suspensions at the edges, keeping the continuous provision of the crystals for deposition. More significantly, the pinning of suspensions at the moving contact line is induced by the gradual pulling of the substrate during the dip-coating process, resulting in the continuous deposition of materials and the formation of a uniform aligned nanoribbons in a large area.

**Fig. 3:**
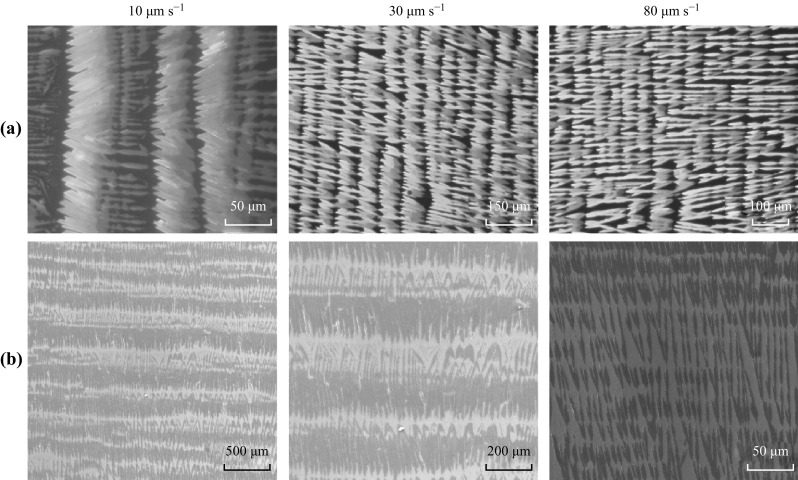
Fluorescence microscope images (**a**) and the corresponding SEM images (**b**) of BPEA nanoribbon arrays on the SiO_2_/Si substrate formed at coating speeds of 10, 30, and 80 μm s^−1^, respectively

**Fig. 4:**
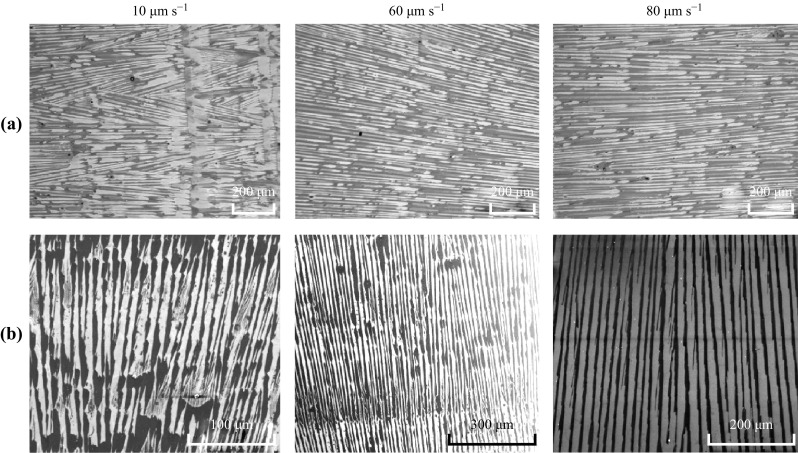
Bright-field optical microscope images (**a**) and the corresponding SEM images (**b**)of TIPS-PEN nanoribbon arrays on the SiO_2_/Si substrate formed at coating speeds of 10, 60, and 80 μm s^−1^, respectively

**Fig. 5:**
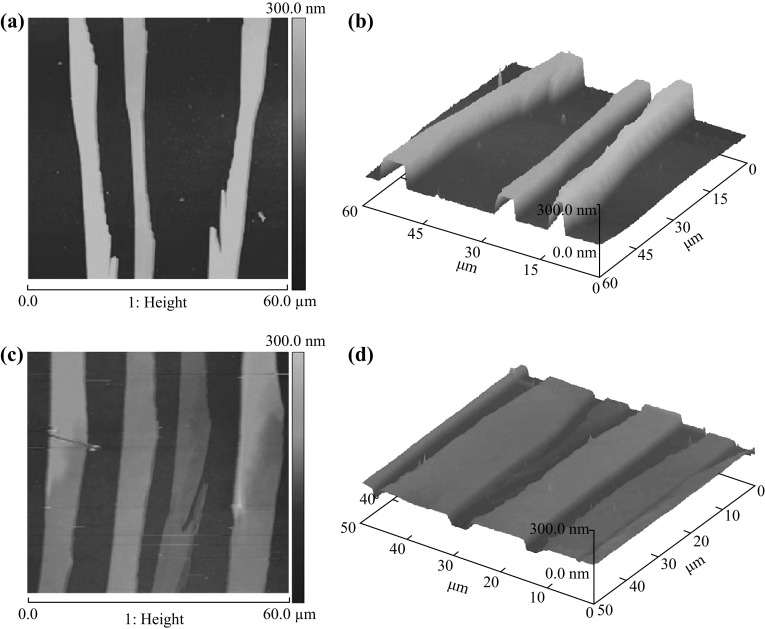
AFM images of BPEA nanoribbon arrays (**a**, **b**) and TIPS-PEN nanoribbon arrays (**c**, **d**) formed at a coating speed of 80 μm s^−1^

It was found that dip-coating speeds played a critical role in controlling the morphologies of nanoribbon arrays. As shown in the optical microscope and SEM images (Figs. 3 and 4), relatively steady contact line can be achieved when lifting rate is faster than 30 μm s^−1^ (for BPEA) and 10 μm s^−1^ (for TIPS-PEN). Nearly continuously aligned nanoribbon arrays with length up to several hundreds of micrometers (for BPEA) or even several millimeters (for TIPS-PEN) were fabricated at an optimum dip-coating speed of 80 μm s^−1^ (for both BPEA and TIPS-PEN). Their morphologies were further investigated by tapping-mode AFM, as shown in Fig. 5. The thickness of BPEA and TIPS-PEN nanoribbons is ~150 and ~50 nm, with a width of 3–5 and 7–10 μm, respectively. All the nanoribbon arrays have faceted edges and smooth surfaces (~1.2 nm), indicating the high quality of the nanoribbon arrays.

In control experiments, when dip-coating speed was increased to be higher than 80 μm s^−1^ (e.g., 120 μm s^−1^), non-continuous and defective nanoribbon arrays with inferior crystallinity were observed (Fig. S2). In addition, if the dip-coating speeds were decreased to be lower than 30 μm s^−1^ for BPEA or 60 μm s^−1^ for TIPS-PEN, periodically aligned short nanoribbons were formed (Figs. 3 and 4). At lower lifting rates, gradual accumulation of organic semiconductors at the contact line made the meniscus too heavy, which induced an increase in the depinning force. As a result, the contact line would slip to a new position, leading to the formation of discontinuous nanoribbon arrays [39, 40]. All these results strongly suggest that the morphologies of nanoribbon arrays mainly depend on the dip-coating speed. In addition, to maintain a proper dip-coating speed, solvents with relatively low boiling point as well as good solubility are preferred, such as the use of dichloromethane as the solvent.

### Aligned Single-Crystalline Organic Nanoribbons for Transistors and LEDs Driving

Well alignment of single-crystalline organic nanoribbons can greatly facilitate OFETs fabrication, since electrodes can be easily deposited perpendicular to the aligned crystals [22, 23], ensuring the high performance and high reproducibility of the devices. OFETs were constructed based on the well-aligned BPEA and TIPS-PEN nanoribbon arrays in a bottom-gate configuration, by depositing Au top-contact source (*S*) and drain (*D*) electrodes through a shadow mask. The dependence of *μ* on the dip-coating speed was systemically investigated. Figures S3a–c and S4a–c show the typical transfer characteristics of nanoribbon array-based OFETs under different dip-coating speeds (i.e., 10–80 μm s^−1^). Twenty devices were tested at each dip-coating speed under ambient environment, and then, the mobilities were calculated. It is shown in Figs. S3d–f and S4d–f that the *μ* of devices obtained at the lowest dip-coating speed (10 μm s^−1^) was almost two orders of magnitude lower than that of the devices obtained at the dip-coating speed of 80 μm s^−1^. These results suggest that nanoribbon arrays with improved crystal qualities can be achieved at higher dip-coating speed.

To further optimize the device performance, we also fabricated OFETs based on the single-crystalline nanoribbon arrays with different channel lengths (Fig. S5). As plotted in Fig. S6a, b, the average mobility gradually increased from 0.3 to 1.8 cm^2^ V^−1^ s^−1^ as the channel lengths increased from 10 to 150 μm for BPEA, while the average mobility increased from 0.1 to 1.35 cm^2^ V^−1^ s^−1^ as the channel lengths increased from 10 to 200 μm for TIPS-PEN. However, with the channel lengths further increased, the mobility began to drop dramatically. In the short-channel regime, the effect of the parasitic contact resistance decreases as channel length increases, thereby enhancing the calculated mobility [41]. With the further increase of channel length, the quantity of crystal defects in the channel will increase, which will in turn impair the device performance [42, 43]. Therefore, appropriate channel length is needed to achieve high-performance devices.

Metallic electrode is regarded as another important factor that may impact the device performance by varying the contact resistance. Figure S6c, d illustrates the electrical characteristics of nanoribbon array-based OFETs with the use of different metallic electrodes, including Au, Ag, Cu, and Al. It is noted that the OFETs with Cu as *S*–*D* electrodes exhibit far superior performance as compared to other kinds of electrodes. This effect can be explained by the low work function of Ag and Al electrodes and the detrimental interpenetration of Ag into the layer of organic semiconductors, which both dramatically degrade the OFETs performance. As for Cu electrodes, a thin Cu_*x*_O interfacial layer can be formed under deposition process. The Cu_2_O layer has an energy band gap of 2.17 eV and an electron affinity of 3.2 eV, indicating a valence band position of about 5.37 eV [44–46]. It is noteworthy that the valance band position of Cu_2_O is higher than Au (5.1 eV), Ag (4.2 eV) and also aligned with the highest occupied molecular orbital (HOMO) levels of BPEA (5.49 eV) [30] and TIPS-PEN (5.34 eV) [47], leading to a striking reduction of the hole-injection barrier and consequently the lower contact resistance.

**Fig. 6:**
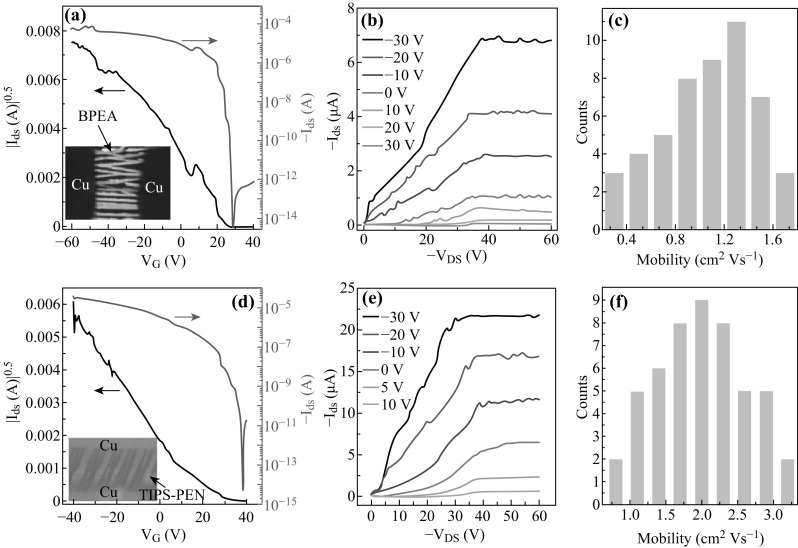
Typical transfer (*V*_DS_ = −50 V) and output characteristics of BPEA (**a**, **b**) and TIPS-PEN (**d**, **e**) nanoribbon array-based OFETs with optimized device configurations on SiO_2_/Si substrates. *Insets* in **a** and **d** show the typical optical images of the BPEA and TIPS-PEN-based OFETs, respectively. Corresponding statistical diagrams of mobilities are shown in **c** and **f** for BPEA and TIPS-PEN nanoribbon array-based OFETs, respectively

At optimized device configurations, 50 devices based on BPEA and TIPS-PEN nanoribbon arrays were examined, respectively. Figure 6 displays the typical device characteristics of the BPEA and TIPS-PEN nanoribbon array-based OFETs on SiO_2_/Si substrates. For BPEA nanoribbon arrays, mobility as high as 2.0 cm^2^ V^−1^ s^−1^ (average *μ* of 1.2 cm^2^ V^−1^ s^−1^), *I*_on_/*I*_off_ > 10^9^, and *V*_T_ about 25 V were obtained (Fig. 6a–c). Notably, this mobility value is higher than that of previously reported BPEA-based OFETs (usually under 1.0 cm^2^ V^−1^ s^−1^ [33, 34]). For TIPS-PEN nanoribbon arrays, mobility as high as 3.2 cm^2^ V^−1^ s^−1^ (average *μ* of 2.0 cm^2^ V^−1^ s^−1^), *I*_on_/*I*_off_ > 10^9^, and *V*_T_ about 10 V were obtained (Fig. 6d–f). The mobility value is also higher than most previously reported TIPS-PEN-based OFETs, which were normally under 3.0 cm^2^ V^−1^ s^−1^ [32, 39]. Moreover, these devices display excellent operating cycle stability with continuous on/off cycles for a period of 1300 and 1500 s for BPEA and TIPS-PEN nanoribbon arrays, respectively (Fig. S7). In addition to the high device performance, it is noteworthy that the deposition area of the single-crystalline organic nanoribbon arrays in this work (50 cm^2^) is much larger than that in the previous reports, in which a small area of lower than 10 cm^2^ was usually demonstrated [39]. Large-area fabrication of the single-crystalline organic nanoribbon arrays offers opportunities for high-performance, low-cost OFET applications.

**Fig. 7:**
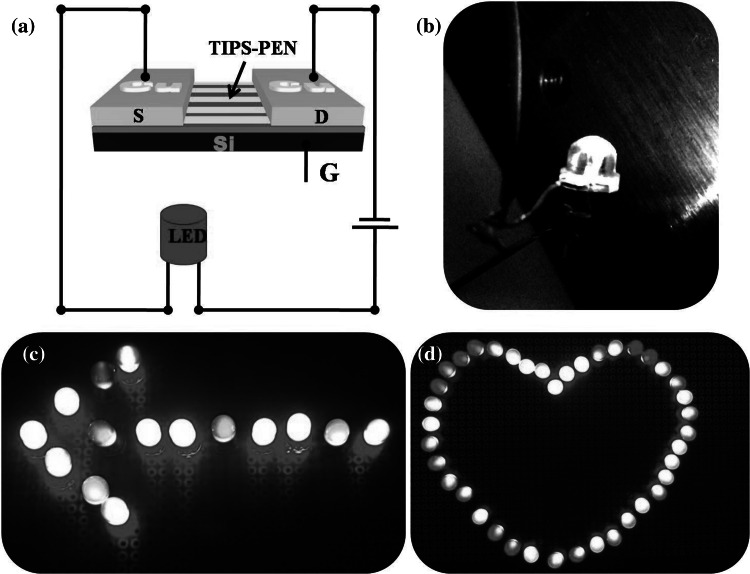
**a** Circuit schematic of the ribbon array-based OFET-LED system. **b** Photograph of one LED driven by the OFET based on TIPS-PEN ribbon arrays. **c**, **d** Show the photographs of the LED pixels driven by the OFET based on TIPS-PEN ribbon arrays

The potential applications of the OFETs in drive circuits were also investigated. OFETs based on TIPS-PEN nanoribbon arrays were used as drivers to control the switching of LEDs. Figure 7a presents the circuit schematic of the nanoribbon array-based OFET-LED system. Figure 7b proves that single OFET has a good current modulation for LED, and the LED can be switched on when the OFET is at the ON state. Also, the OFETs are capable of modulating the light emission of multiple LEDs, forming the different emission patterns (Fig. 7c, d). In addition, the visual brightness of LEDs can be readily controlled by tuning the gate voltages of the OFETs. This result demonstrates that the large-area aligned single-crystalline organic nanoribbon array-based OFETs are promising for electronic applications, such as driving LED pixels, sensors, and basic logic circuits [48–51].

## Conclusions

We successfully fabricated large-area single-crystalline organic nanoribbon arrays via a simple solution-processed dip-coating method. During the growth process, organic molecules tended to crystallize at the three-phase contact line. Through carefully controlling the coating speed, continuous and well-aligned organic nanoribbon arrays could be obtained. Moreover, we demonstrated that the performance of the OFETs based on the organic nanoribbon arrays could be remarkably improved by optimizing the channel lengths and using appropriate metallic electrodes. Under the optimal device configurations, OFETs based on aligned BPEA single-crystalline nanoribbon arrays gave rise to a mobility up to 2.0 cm^2^ V^−1^ s^−1^, while OFETs based on aligned TIPS-PEN nanoribbon arrays achieved a maximum mobility of 3.2 cm^2^ V^−1^ s^−1^. These values are superior or comparable to the best results reported for these two small molecules, while the growth area of the aligned nanoribbon arrays (50 cm^2^) represents the largest size reported up to now. Moreover, the organic nanoribbon array-based OFETs exhibit long-time cycle stability, enabling the control of light emission of different pixel patterns of LEDs. This method offers a means for the fabrication of large-area, aligned single-crystalline organic nanoribbons. Their applications for OFETs and LED driving open up opportunities for future high-performance, low-cost organic electronic and optoelectronic devices.

## Electronic supplementary material

Below is the link to the electronic supplementary material.

Supplementary material 1 (PDF 1194 kb)
