# Supplementary material for: Controlled Growth of Large-Area Aligned Single-Crystalline Organic Nanoribbon Arrays for Transistors and Light-Emitting Diodes Driving
Source: Nanomicro Lett. 2017 Aug 16;9(4):52. doi: 10.1007/s40820-017-0153-5 (PMC6199044; doi:10.1007/s40820-017-0153-5)
Supplement: Supplementary file 1 — Supplementary material 1 (PDF 1194 kb) [file 40820_2017_153_MOESM1_ESM.pdf]

Supporting Information for

Controlled Growth of Large-Area Aligned Single-Crystalline Organic Nanoribbon Arrays for Transistors and Light-Emitting Diodes Driving

Wei Wang[1], Liang Wang[1], Gaole Dai[1], Wei Deng[1], Xiujuan Zhang[1], Jiansheng Jie[1,] *, Xiaohong Zhang[1]

[1] Institute of Functional Nano & Soft Materials (FUNSOM), Jiangsu Key Laboratory for Carbon-Based Functional Materials & Devices, Soochow University Suzhou Jiangsu 215123, People's Republic of China

*Corresponding author. E-mail: jsjie@suda.edu.cn

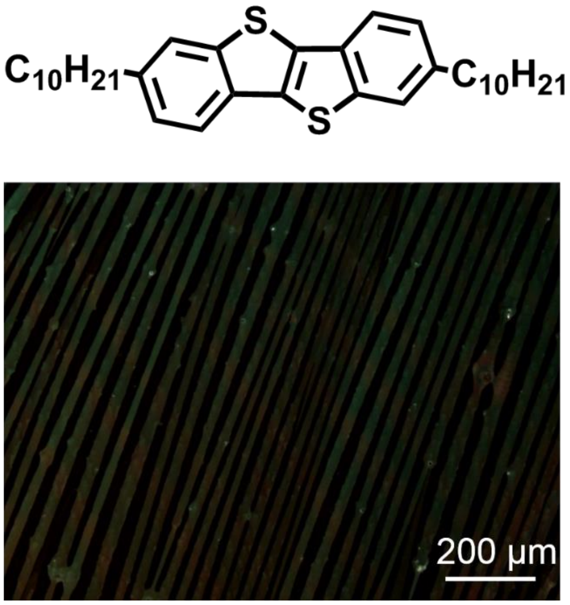

Fig. S1 Molecular structure of the C10-BTBT and corresponding cross-polarized microscope image of ribbon arrays by using dip-coating method

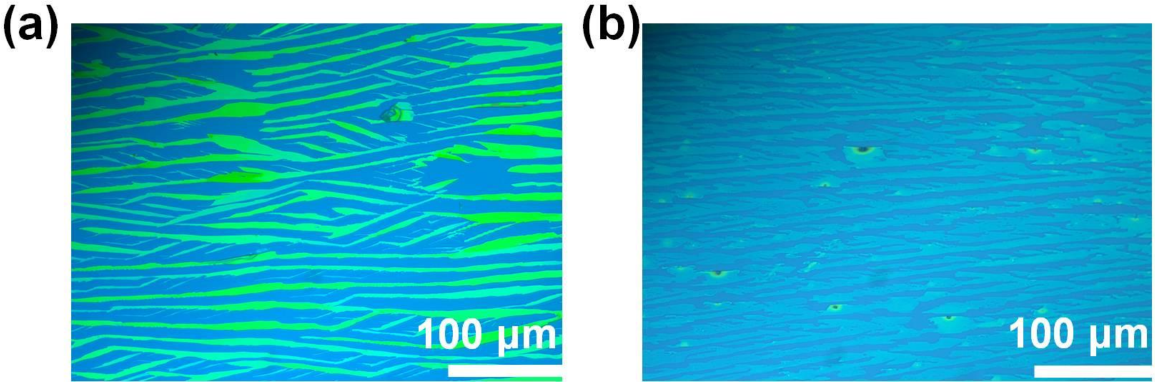

Fig. S2 Fluorescence microscope images of **a** BPEA and **b** TIPS-PEN nanoribbon arrays on the SiO_2/Si substrates formed at a coating speed of 120 μm s^{-1}

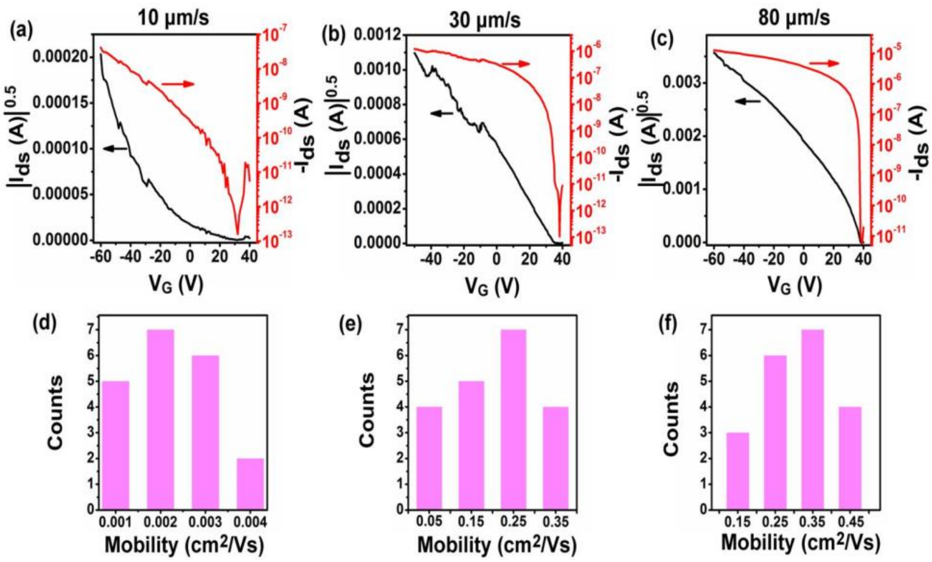

Fig. S3 Typical transfer characteristics (V_{DS} = -50 V) of the OFETs based on BPEA nanoribbon arrays fabricated at different coating speeds of 10, 30, and 80 μm s^{-1} (**a, b, c**), respectively, on the SiO_2/Si substrates. **d-f** The corresponding statistical diagrams of mobilities. The OFETs use u as *S* and *D* electrodes and have *L* of 40 μm

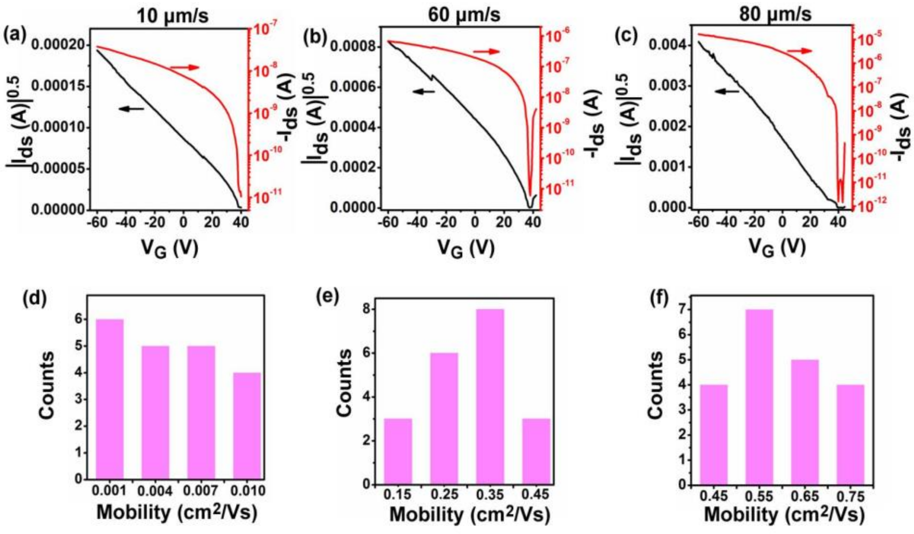

Fig. S4 Typical transfer characteristics (V_{DS} = -50 V) of OFETs based on TIPS-PEN nanoribbon arrays fabricated at different coating speeds of 10, 60, and 80 μm s^{-1} (**a, b, c**), respectively, on the SiO_2/Si substrates. **d-f** The corresponding statistical diagrams of mobilities. The OFETs use Au as *S* and *D* electrodes and have *L* of 40 μm

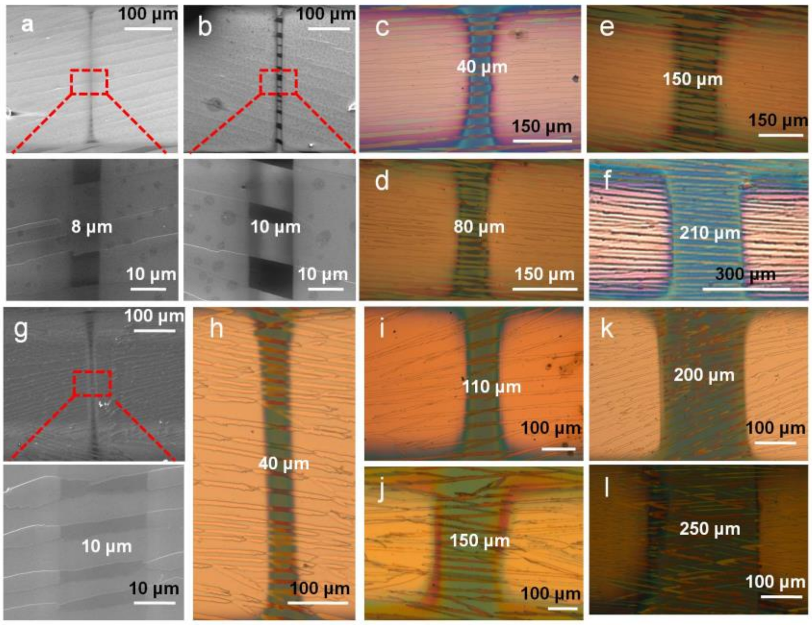

Fig. S5 Optical microscope images of the OFETs based on **a-f** TIPS-PEN nanoribbon arrays and **g-l** BPEA nanoribbon arrays fabricated at coating speeds of 80 μm s^{-1}, respectively, with different channel lengths

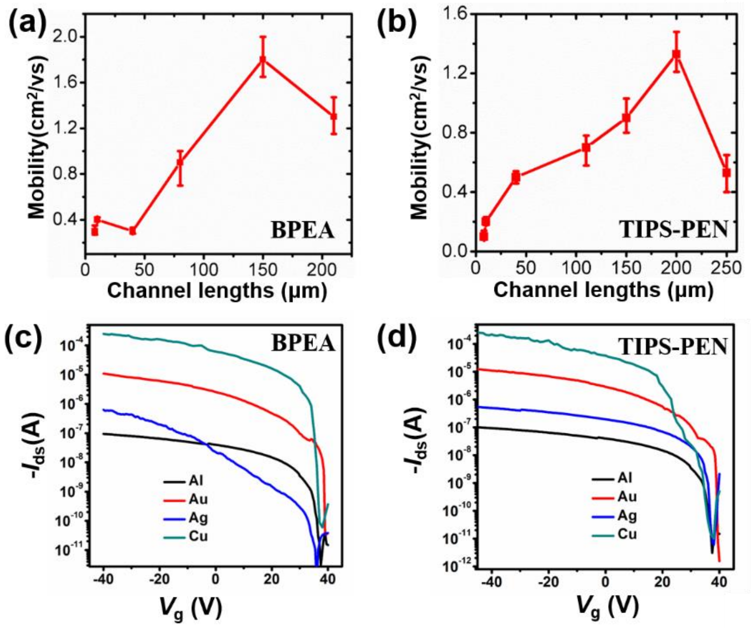

Fig. S6 Average charge-carrier mobility of the OFETs fabricated from BPEA (**a**) and TIPS-PEN (**b**) nanoribbon arrays at a coating speed of 80 μm s^{-1} with different channel lengths. The typical transfer characteristics (V_{DS} = -50 V) of OFETs based on the BPEA (**c**) and TIPS-PEN (**d**) nanoribbon arrays fabricated at a coating speed of 80 μm s^{-1} with different metallic *S* and *D* electrodes

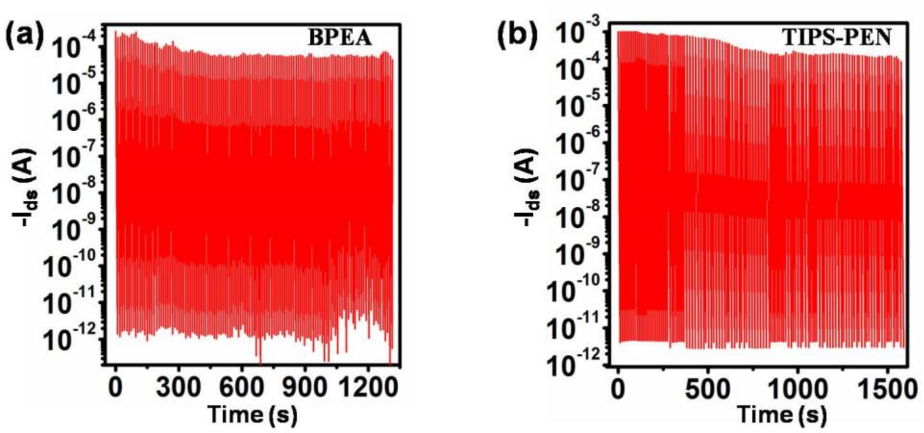

Fig. S7 Cycle stability of BPEA (**a**) and TIPS-PEN (**b**) nanoribbon array-based OFETs with a continuous ON (V_G = -60 V) and OFF (V_G = 38 V) cycles
